# The Role of Relative Deprivation and Attribution Style in the Relationship between Organizational Fairness and Employees’ Service Innovation Behavior

**DOI:** 10.3390/bs12120506

**Published:** 2022-12-12

**Authors:** Zhao Li, Wangbing Liang, Yinggang Bao, Ruili Zhang

**Affiliations:** 1 Tourism College, Gansu Tourism Development Academy, Northwest Normal University, Lanzhou 730070, China; 2 Economics College, Lanzhou University, Lanzhou 730070, China

**Keywords:** organizational fairness, service innovation behavior, relative deprivation, attribution style, social cognitive theory

## Abstract

The basis of organizational innovation is employee innovation, which is of great significance for organizations to gain a competitive advantage. At present, the research on the influencing factors of employee service innovation behavior is increasing. This study, based on the social cognitive theory, with relative deprivation as the mediator and attribution style as the moderator, explores the mechanism of the effect of organizational fairness on employee service innovation behavior. Taking 342 employees of service-oriented enterprises as the subjects of investigation, this paper empirically tests the theoretical model by using Amos and SPSS. The results indicated the following, organizational fairness was positively related to employees’ service innovation behavior. Relative deprivation partially mediated the relationship between organizational fairness and service innovation behavior. An external attribution style positively moderated the relationship between relative deprivation and employee service innovation behavior. An external attribution style also positively moderated the mediation effect of the relationship between organizational fairness and service innovation behavior. The internal attribution style negatively moderated the relationship between relative deprivation and employee service innovation behavior. The internal attribution style also negatively moderated the mediation effect of the relationship between organizational fairness and service innovation behavior. The conclusion of this study has managerial implications on how to promote employee service innovation behavior in service-oriented enterprises.

## 1. Introduction

At present, under the influence of COVID-19, service-oriented enterprises, such as hotels and tourism, are facing great crises and challenges [1]. At the same time, the proportion of the service industry in the national economy has been increasing, and services have become the most active factor and the most important force in the social economy. In the era of the service economy, the market competition of service-oriented enterprises is increasingly fierce. In addition, with the continued diversification and individualization of customer needs, service-oriented enterprises are facing tremendous pressure and challenges when providing services to customers [2]. Employee innovation is the foundation of organizational innovation, and it is also the source and motivating force of organizational survival and development. It is of great significance to improve the competitiveness of enterprises and guarantee customer loyalty [3]. For service-oriented enterprises, how to make employees deal with problems flexibly and solve problems creatively so as to ensure the quality of service and improve customer loyalty and corporate image has become a matter of urgent concern [4]. Most of the posts of employees in service-oriented enterprises have practical attributes. The service innovation behavior of employees in the process of work can promote the continuous innovation of enterprise service forms, thus enhancing the ability of enterprises to meet the needs of customers to ensure that enterprises can maintain a competitive advantage in market competition [5]. Service innovation behavior is usually not in the service scope stipulated by the post-duty requirement, and the employees often need to undertake certain responsibilities and risks when carrying out the service innovation in the process of work; previous studies have found that both individual factors and situational factors have an impact on individual service innovation behavior [6]. Therefore, it is an important issue that cannot be neglected for service-oriented enterprises to discover the factors that hinder the service innovation behavior of employees and stimulate the service innovation behavior of employees.

Research on employee service innovation behavior is one of the hot spots in the field of human resource management. In the process of providing services, employees generate new ideas to solve problems and put them into practice, thus promoting organizational innovation, which plays an important role in promoting the core competitiveness of enterprises [7]. How to stimulate the service innovation behavior of the individual in the enterprise is always an important proposition in the organization management, but in some cases, even with the encouragement and promotion of the organization, many employees are still reluctant to experiment with service innovation [8]. The research on the influencing factors of service innovation behavior can be summarized in two aspects: individual factors and situational factors. The former includes self-knowledge [9], personality traits [10], intrinsic motivation [11] and other individual subjective factors. The latter includes organizational culture [12], leadership style [13], organizational structure [4], and other situational factors.

As a subjective feeling of employees, organizational fairness depends on their evaluation of fairness [14]. Organizational fairness is an important index to measure the management level and competitiveness of enterprises, and research shows that organizational fairness can not only affect the attitude and behavior of employees [15], but it is also closely related to the stress and emotional reaction of employees [16]. A fair organizational environment can increase employee satisfaction and performance levels, evoke positive emotional perceptions of employees, and thus have a more positive behavioral response to the organization [17]. When employees feel that the organizational environment is unfair, they will think that their interests have not been properly protected and respected, thus resulting in a negative emotional reaction, having a disadvantageous influence on the employee’s work behavior and the organization’s unfolding work [18]. However, there are few studies on the mechanism of the effect of organizational fairness on the service innovation behavior of employees in service-oriented enterprises; then, as an important subjective feeling of the organization’s staff—organizational fairness—whether it will affect the work behavior of the organization employees. Specifically, for the employees of service-oriented enterprises, whether organizational fairness is a factor affecting the service innovation behavior of employees and what is the mechanism and boundary condition of the effect of organizational fairness on the service innovation behavior are topics worthy of further discussion both in theory and in practice.

The research on the intermediary mechanism of service innovation behavior by scholars mainly takes two paths: external environmental conditions and individual characteristics; the variables involved included intrinsic motivation [19], self-efficacy [20], work autonomy [21], organizational support [22], and so on. However, the research on the effect of organizational fairness on the service innovation behavior of employees in service-oriented enterprises from the perspective of relative deprivation has not been discussed yet. Organizational fairness is one of the important bases for employees to perceive the organizational environment, and it is also an important factor influencing employees’ psychological state and beliefs [23]. Employees’ perceptions of the fairness of the organizational environment and the resulting psychological states and beliefs have an impact on their behavior [24,25]. Relative deprivation expresses an individual’s perception of their own disadvantage, as well as the negative emotions such as anger and unhappiness that result from comparison with the reference group [26]; relative deprivation may occur when employees feel that they have been treated unfairly in the organization through comparison, and then affect the attitude and behavior of employees. Therefore, this paper suggests that employees’ relative deprivation may play a mediation role between organizational fairness and employees’ service innovation behavior. Furthermore, although organizational fairness is prone to changes in employee mood and psychology, the extent to which it affects outcomes is often influenced by individual idiosyncrasies [27]. Attributional style is a cognitive style that can reflect personality traits with individual differences and relative stability [28]. Individuals who tend to attribute to external are more likely to attribute perceived inequities in the work environment to external environmental causes, such as organizational system inequities, resulting in a greater psychological gap and psychological imbalance, the expression of negative emotions is more prominent, which has a negative impact on their behavior [29]. At present, there is still a gap in the research on the effect of attribution on service innovation behavior in service-oriented enterprises; in order to make up for this deficiency, this study will discuss the moderation effect of attribution style on the relationship between relative deprivation and service innovation behavior in service-oriented enterprises.

Based on social cognitive theory, this study attempts to explore the effect of organizational fairness on the service innovation behavior of employees in service-oriented enterprises from the perspective of employees. By focusing on the psychological perception and attribution of organizational fairness in the workplace of employees in service-oriented enterprises, to explore the internal mechanisms of this process (the mediation effect of relative deprivation) and whether there is a boundary effect (the moderation effect of the attributional style). In order to theoretically make up for the deficiency of the research on service innovation behavior of employees in service-oriented enterprises in the field of human resource management, and also provide theoretical guidance for managers of service-oriented enterprises to improve their employees’ organizational fairness and inspire their service innovation behavior by improving their working environment, working atmosphere and improving their organizational system.

## 2. Literature Review and Hypothesis Development

### 2.1. Organizational Fairness and Service Innovation Behavior

On the basis of the reasonable utilization of organizational resources, the service innovation behavior of employees means that in order to improve customer satisfaction and service quality, employees should give full play to their own advantages and abilities, the process of implementing innovative ideas such as new ideas and approaches to customer service [30]. From this perspective, a small number of studies have measured employee service innovation behavior as a multi-dimensional variable; for example, Kleysen measured employee service innovation behavior in five dimensions: finding opportunities, generating innovative ideas, evaluating innovative ideas, supporting innovative ideas, and applying practices [31]. Most research treats employee service innovation behavior as a single-dimension variable based on the definition of process perspective, mainly measuring the degree of realization of employee innovation behavior. Among them, Scott and Bruce’s definition and measurement of individual innovation behavior are widely accepted and applied [32]. At present, the research on the influencing factors of employee service innovation behavior mainly includes two aspects: individual factors and situational factors. The former mainly includes the influence of individual cognitive, psychological, and behavioral factors [9,10,11], and the latter mainly includes organizational climate, support, interpersonal interaction, and other factors [4,12,13].

Previous studies have explored the factors that influence employees’ service innovation behavior, but the existing research on the impact of organizational fairness on service-oriented enterprises’ employees’ service innovation behavior is insufficient. Organizational fairness refers to the subjective feelings that employees form by comparing their past selves with their present selves and comparing their own inputs and outputs with those of other colleagues [33]. The internal environment of an organization is an important foundation for the survival and development of an organization, and the psychological environment of employees is an important component of the internal environment of an organization and has a significant impact on employee motivation and behavior [34]. Employees with a low sense of organizational fairness are prone to negative emotions such as jealousy, which affect their spirit of cooperation and dedication, and are more likely to engage in negative behaviors in their work performance [35]. Guo et al. found that employees’ organizational fairness would have a negative impact on their anti-productive behavior [36]. RH Moorman’s study found that organizational fairness has a positive effect on employees’ OCB [37]. Fo Walumbwa et al. found that organizational fairness has a positive effect on employees’ voluntary learning behavior [38].

The social cognitive theory emphasizes the role of individual motivation and holds that an individual’s environment will influence their cognition. Individuals take positive actions to remove the limitations of the environment and achieve the expected results through observation, learning, and self-regulation [39]. A fair organizational environment can enable employees to have a sense of trust and respect so as to improve employee satisfaction and work enthusiasm and encourage employees to take some actions beyond the requirements of their job duties to help improve performance [39]. Due to the potential risk of service innovation behavior leading to employee mistakes, only under appropriate conditions can employees be willing and able to put into practice new ideas, new methods, and other innovative ideas of customer service [40]. Individuals who experience high organizational fairness have a more solid relationship with the organization and have higher job satisfaction and enthusiasm, so it is easier to stimulate their new ideas to improve work processes and provide innovative services. A fair organizational environment is also conducive to strengthening the employees’ sense of trust in the organization so that they can more often dare to take some actions beyond the requirements of their job responsibilities to improve performance and put innovative ideas into practice [41]. Therefore, this paper proposes the following hypothesis:

**Hypothesis 1 (H1).**  *Organizational fairness has a significant positive effect on employees’ service innovation behavior*.

### 2.2. Mediation Effect of Relative Deprivation

Relative deprivation is a kind of cognitive and emotional experience, which refers to a subjective cognitive and emotional experience in which individuals or groups feel that they are at a disadvantage in comparison with reference objects, leading to negative emotions such as anger and dissatisfaction [26]. The existing research shows that the relative deprivation of employees is mainly due to the interaction of horizontal comparison with colleagues and vertical comparison of individual self [42]. When individuals have a sense of relative deprivation, it will further lead to negative emotions and negative behaviors [43]. This variable will have an impact on employees’ internal motivation and is an important antecedent variable to predicting employees’ attitudes and behaviors.

This paper argues that relative deprivation plays a mediation role between organizational fairness and employee service innovation behavior:

Firstly, organizational fairness has a negative impact on relative deprivation. According to the social cognitive theory, the environment of an individual will have an impact on their cognition [44]. On the one hand, an unfair organizational environment makes employees more sensitive and increases their distrust in the organization and colleagues [45]. At the same time, an unfair organizational environment will cause employees to feel at a disadvantage in the process of comparison with others within the organization and with their own expected gains and reality, which will lead to employee dissatisfaction within the organization and hostility to other colleagues, leading to an increased sense of individual relative deprivation [46]. On the other hand, when employees who perceive the unfair environment of the organization are in a closed organizational environment or lack the ability to intervene and change the status quo, it will further lead to employees feeling marginalized in the organization [47]. Although individuals are organizational employees, they feel isolated from the organization, which reduces employees’ recognition and attachment to their organization. This psychological feeling may also be expanded subjectively by individuals, affecting their daily work in the organization. Based on this, according to the individual’s perception of the organizational situation formed by perceived organizational unfairness, organizational employees have psychological hints of hostility to their interpersonal relationships and perceptions of a poor working environment, resulting in their increased sense of relative deprivation in the workplace.

Secondly, the perception of unfairness in the organizational environment increases the employees’ sense of relative deprivation, which further leads to reduced employee service innovation behavior. The social cognitive theory emphasizes the self-regulation of individuals and the interaction between individuals and the environment under the active role [48]. On the one hand, if the change in the employees’ psychological state caused by an unfair organizational environment is due to the comparison with others in the organization, it will have a negative impact on their relationship with colleagues, resulting in more social avoidance [49]. Employees feel that their situation in the organization is worse, generate angry or resentful emotional reactions, reduce their interaction with the environment, and thus reduce their activity in the organization. On the other hand, an increased sense of relative deprivation may reduce employee loyalty and their sense of belonging in the organization, even disgust with the organization. They no longer regard themselves as part of the organization and seldom produce or stop their service innovation behavior [50]. In addition, when employees lack the ability to intervene and change the unfair situation in the organization, they will vent their sense of deprivation by taking negative work or even anti-productive behaviors [51]. Due to an increased sense of relative deprivation, employee enthusiasm for work and a sense of belonging in the organization are adversely affected, and they no longer actively engage in activities conducive to the organization, leading to reduced service innovation behavior.

To summarize, this paper believes that when employees of service-oriented enterprises perceive unfairness in the organizational environment, they will have negative emotions such as dissatisfaction and alienation towards the organization and even other colleagues. Employees perceive the deterioration of the organizational atmosphere and internal environment, resulting in an increase in their sense of relative deprivation, reducing their positive behavior and leading to the reduction or cessation of service innovation behavior. Therefore, this paper proposes the following hypothesis:

**Hypothesis 2 (H2).**  *Relative deprivation plays a mediation role between organizational fairness and service innovation behavior*.

### 2.3. Moderation Effect of Attribution Style

Although relative deprivation may lead to reduced employee service innovation behavior, different employees have different cognitive processes for the causes of the results; different individuals have different perceptions and responses to the same adverse environment [52]. In order to further explore the boundary conditions under which relative deprivation affects employee service innovation behavior, the attribution style is introduced to explore its moderation effect on the above relationship. The attribution style is a cognitive style that refers to the tendency of individuals to make consistent explanations for all the events that occur in their lives. The attribution style has been formed earlier and is relatively stable, which may play a regulatory role in the relationship between negative experience and experience results in employees’ work [53]. Employees can be classified according to the different attribution methods of different employees [54]: when employees tend to use the external attribution method when they perceive unfairness in the internal environment of the organization, they will attribute it to external reasons, such as unfair organizational policies, and thus tend to make stronger responses and generate stronger negative emotions. Employees who tend to attribute their sense of unfairness to internal reasons, such as their lack of effort or personality or ability problems, will have weak emotions and reactions.

This study believes that external attribution positively moderated the relationship between relative deprivation and employee service innovation behavior, while internal attribution negatively moderated the relationship between relative deprivation and employee service innovation behavior.

As a cognitive and emotional experience of individuals, relative deprivation is highly subjective in employees’ perception of it. Individuals with different attribution methods have different intensities of behavior and emotional reactions after generating relative deprivation. Compared with employees who prefer internal attribution, employees who prefer external attribution are more likely to attribute the reason why they feel disadvantaged by comparing the reference object to the external environment, thus causing them to have negative emotions such as dissatisfaction with the organization and even colleagues, which leads to negative behaviors. According to social cognitive theory, individuals can activate different environmental responses through their own subjective characteristics, such as personality, social roles, etc. [48]. Individuals who tend to attribute to the external are more likely to attribute the cause of adverse cognition and negative emotions to the external environment and are more likely to make a strong response. Therefore, the negative problems caused by relative deprivation are more obvious, and their service innovation behavior will be greatly affected. Therefore, this paper proposes the following hypothesis:

**Hypothesis 3a (H3a).**  *External attribution can moderate the negative relationship between relative deprivation and employee service innovation behavior. The more employees tend to external attribution, the stronger the negative relationship between relative deprivation and employee service innovation behavior*.

On the contrary, employees who prefer the internal attribution style are more likely to attribute the causes of adverse cognition and negative emotions to their own internal causes and will not have strong negative emotions towards the organization and colleagues, thus reducing the impact of relative deprivation on employees. Their service innovation behavior is less affected by relative deprivation and may even be strengthened due to their own internal reasons. Therefore, this paper proposes the following hypothesis:

**Hypothesis 3b (H3b).**  *Internal attribution can moderate the negative relationship between relative deprivation and employee service innovation behavior. The more employees tend to internal attribution, the weaker the negative relationship between relative deprivation and employee service innovation behavior*.

### 2.4. Moderated Mediating Effect of Attribution Style

**Figure 1:**
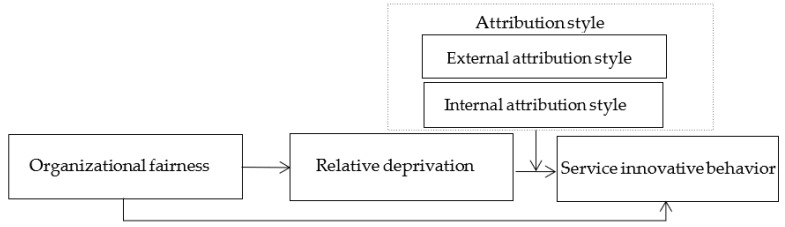
Conceptual model.

Combining H2, H3a, and H3b, this paper proposes a moderated mediating effect model (Figure 1). It can be seen from the above that relative deprivation plays a mediation role in the relationship between organizational fairness and employee service innovation behavior, and external attribution positively moderate the relationship between relative deprivation and employee service innovation behavior, while internal control attribution negatively moderates the relationship between relative deprivation and employee service innovation behavior. It is further inferred that the intermediary effect of relative deprivation between organizational fairness and employee service innovation behavior may also be affected by attribution style.

When employees tend toward the external attribution style, employees with a low sense of organizational fairness are more likely to feel disadvantaged when compared to a reference object, resulting in a sense of relative deprivation, which leads to reduced service innovation behavior [55]. For employees with a high sense of organizational fairness, even if they do not feel that they are at a disadvantage by comparing with the reference object, they may also adjust their service innovation behavior because they tend toward an external attribution style. That is to say, the tendency towards external attribution style will strengthen the negative relationship between relative deprivation and employee service innovation behavior so that the sense of organizational fairness can enhance the effect of weakening employee service innovation behavior through relative deprivation [56]. Therefore, this paper proposes the following hypothesis:

**Hypothesis 4a (H4a).**  *External attribution style positively moderates the intermediary role of relative deprivation between organizational fairness and service innovation behavior*.

When employees are inclined toward an internal attribution style, employees with a low sense of organizational fairness feel that they are at a disadvantage by comparing with the reference object and thus have a sense of relative deprivation; they may also be willing to adjust themselves to meet organizational requirements because they are inclined to the internal attribution style, thus showing a certain degree of service innovation. In other words, the tendency towards internal attribution style will weaken the negative relationship between the sense of relative deprivation and employee service innovation behavior so that the sense of organizational fairness will weaken the effect of employee service innovation behavior through the sense of relative deprivation [57]. Therefore, this paper proposes the following hypothesis:

**Hypothesis 4b (H4b).**  *Internal attribution style negatively moderates the intermediary role of relative deprivation between organizational fairness and service innovation behavior*.

## 3. Methodology

### 3.1. Data Collection and Research Objects

In this study, the data were collected using a questionnaire. During the preparation of the questionnaire, the mature scale in high-level journals was selected, and appropriate adjustments were made in combination with the specific research in this paper to ensure the reliability and validity of the questionnaire. The Likert 5 scale was used for all items.

Specifically, for the measurement of organizational fairness, refer to the scale [57] developed by Ambrose and others, including 6 items: “Generally speaking, the company treats you fairly”; “My work arrangement is fair”; “The superior’s work decisions are made in an unbiased way”; “All work decisions are applied consistently to all relevant employees”; “My superiors will treat me sincerely for decisions involving my work”; “When making decisions about my work, my superiors will give me explanations that I can understand”.

For the measurement of relative deprivation, refer to Cho et al. [58]’s cognitive emotional R D dual dimensional structure model, including four items: “Compared with the surrounding colleagues, you feel your salary is relatively low”; “Compared with your expectation or the past, you feel your salary is relatively low”; “Compared with the colleagues around you, the organization gives you less resources (status, reputation, rights, etc.)”; “Compared with your expectation or the past, the organization gives you less resources (status, reputation, rights, etc.)”.

For the measurement of service innovation behavior, refer to the scale [7] prepared by Scott and Bruce, which contains 4 items: “I am good at searching for new technologies, processes, technologies /product ideas in my work”. “In my work, I will generate creative ideas”; “I will investigate and seek the funds needed to implement new ideas”; “I will make appropriate plans and timetables for implementing new ideas”.

For the measurement of attribution style, refer to the Multidimensional Multi Attribution Causality Scale [59] prepared by Lefcourt et al. This scale includes four dimensions: ability, effort, situation, and luck. Among them, ability and effort are internal attribution styles, while situation and luck are external attribution styles. It includes 8 items, 4 of which measure the internal attribution style: “In my opinion, getting along with others is a skill”; “In my experience, there is a direct relationship between lack of friendship and social incompetence”; “It takes effort to maintain friendship”; “As far as I am concerned, the success of making friends depends on how much I have done”. Four items measure external attribution style: “No matter what I do, some people just do not like me”; “Even when I do not want to associate with others, some people can make me have a good time”; “Accidents often account for a large proportion of the discord between friends”; “According to my experience, making friends is mostly a matter of luck”.

In this study, employees of service-oriented enterprises, such as hotels and exhibition enterprises, were investigated. In order to reduce the impact of common method bias, the survey method of multiple time periods and multiple data sources was adopted. Before the formal survey, a small-scale pre-survey was carried out, and the opinions and suggestions expressed on the survey items were adjusted according to the questionnaire respondents so as to make the survey items of the questionnaire easier to understand. Formal research is conducted online and offline at the same time. The online respondents were employees of two hotels that supported this study. Using the method of random sampling, offline surveys were conducted on a multiple-time and multiple-data source survey on employees of service enterprises such as hotels and exhibition enterprises in Taiyuan, China. The survey was conducted from July to August 2022. A total of 384 valid questionnaires were collected by the two methods. On this basis, the researcher further eliminated the questionnaires with obvious filling errors and serious data missing and finally obtained 342 valid questionnaires, with an effective rate of 89.1%.

### 3.2. Sociodemographic Data

In the valid questionnaire, the male proportion was 37.7%, and the female proportion was 62.3%. The overall age of the employees is relatively young, including 37.1% aged 18–25, 30.5% aged 26–30, 21.6% aged 31–40, and 10.8% aged over 40. Most of them have a junior college education, among which 24.6% are at senior high school level or below, 49.1% are at junior high school level, 19.6% are at the undergraduate level, and 6.7% are at the Master’s level or above. Work experience of less than one year accounted for 32.7%, 1–3 years accounted for 31.6%, 3–5 years accounted for 21.9%, and more than 5 years accounted for 13.7%. This sample is in line with the basic characteristics of employees in service-oriented enterprises and has a certain representativeness.

## 4. Analysis and Results

### 4.1. Reliability and Validity Test

First, SPSS was used to test the reliability. The results showed that the standardized load coefficients of organizational fairness, relative deprivation, service innovation behavior, internal attribution style, and the external attribution style were bigger than 0.6, Cronbach’s α were 0.92, 0.866, 0.883, 0.77, and 0.723, respectively, which indicates that the reliability of the research data is great. Secondly, the arithmetic square root of the extracted value of the average variance of each major variable is bigger than the correlation coefficient between this variable and other variables, indicating that the major variables in this study have good discriminant validity. In addition, the extracted value AVE of variance of each major variable was bigger than 0.5, and the combined reliability (CR) of each variable was bigger than 0.7, indicating that each major variable in this study had good aggregation validity. See Table 1 for details.

### 4.2. Correlation Analysis

It can be seen from the correlation analysis that the statistical relationship between the variables is strong, which provides preliminary evidence for subsequent research. There is a significant negative correlation between organizational fairness and relative deprivation, a significant positive correlation between organizational fairness and service innovation behavior, and a significant negative correlation between relative deprivation and service innovation behavior. See Table 2 for details.

### 4.3. Hypothesis Testing

#### 4.3.1. Testing of Main Effect

The path coefficient and fitting index of the model are shown in Table 3. When organizational fairness affects service innovation behavior, the value of the standardized path coefficient is 0.439 > 0, and this path shows a significant level of 0.01 (z = 3.258, *p* = 0.001 < 0.01), which indicates that organizational fairness will have a significant positive impact on service innovation behavior, and the hypothesis H1 is supported.

#### 4.3.2. Intermediary Effect Testing

**Figure 2:**
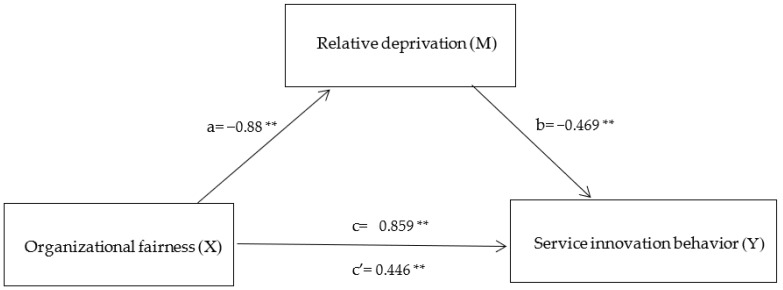
Mediation effects model Note: ** *p* < 0.01; a is effect of Organizational fairness on Relative deprivation; b is effect of Relative deprivation on Service innovation behavior; c’ is direct effect of Organizational fairness on Service innovation behavior; c is total effect of Organizational fairness on Service innovation behavior.

Firstly, when organizational fairness affects relative deprivation, the standardized path coefficient value is −0.923 < 0, and this path shows a significant level of 0.01 (z = −23.421, *p* = 0.000 < 0.01), which indicates that organizational fairness will have a significant negative impact on relative deprivation. When relative deprivation affects service innovation behavior, the standardized path coefficient value is −0.441 < 0, and this path shows a significance of 0.01 level (z = −3.269, *p* = 0.001 < 0.01), which indicates that relative deprivation will have a significant negative impact on service innovation behavior, so the hypothesis H2 is supported. At the same time, Bootstrapping analysis technology is used to further verify the mediation effect of psychological security, as shown in Table 4. The mediation effect analysis involves three models, respectively: Service innovation behavior = 0.442 + 0.859 × organizational fairness, relative deprivation = 5.686 − 0.880 × organizational fairness, service innovation behavior = 3.111 + 0.446 × organizational fairness −0.469 × relative deprivation. Table 5 and Figure 2 show that the direct effect of perceived service innovation behavior of organizational fairness through relative deprivation is 0.446 **, with a 95% confidence interval of [0.018, 0.666], excluding no zero. Therefore, H2 is assumed to be further supported. In addition, because the intermediary effect value is the same as the direct effect value, the sense of relative deprivation plays the part of the intermediary role between organizational fairness and service innovation behavior.

#### 4.3.3. Moderation Effect Testing

**Figure 3:**
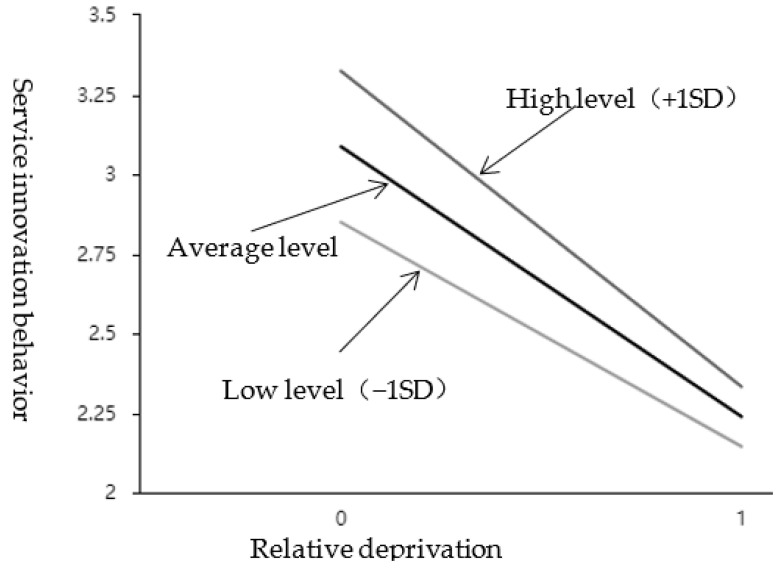
The effect of interaction between RD and EAS on SIB.

As shown in Table 6, the interaction item between relative deprivation and external attribution style presents a significant (t = −2.298, *p* = 0.024 < 0.05) and has a negative impact (β = −0.135, *p* < 0.05). It means that when relative deprivation affects service innovation behavior, the moderation variable (external attribution style) has a significant difference in the extent of influence at different levels. See the simple slope diagram in Figure 3. Divide the scores of the external attribution style into two groups according to high (M + 1SD) and low (M − 1SD). Perform a simple slope analysis. As shown in Figure 3, the top line represents the relationship between relative deprivation and service innovation under a high external attribution style; the bottom line represents the relationship between relative deprivation and service innovation behavior under a low external attribution style; the middle line is the average level. It can be seen that the attributive style of employees’ external is at a low level, and the relationship curve between relative deprivation and employees’ service innovation behavior is gentler, and the slope is smaller; When the attributive style of employees’ external is at a high level, the relationship curve between relative deprivation and employees’ service innovation behavior is steeper, and the slope is bigger. The positive moderation effect of external control attribution was further tested; that is, H3a was further supported by empirical data.

**Figure 4:**
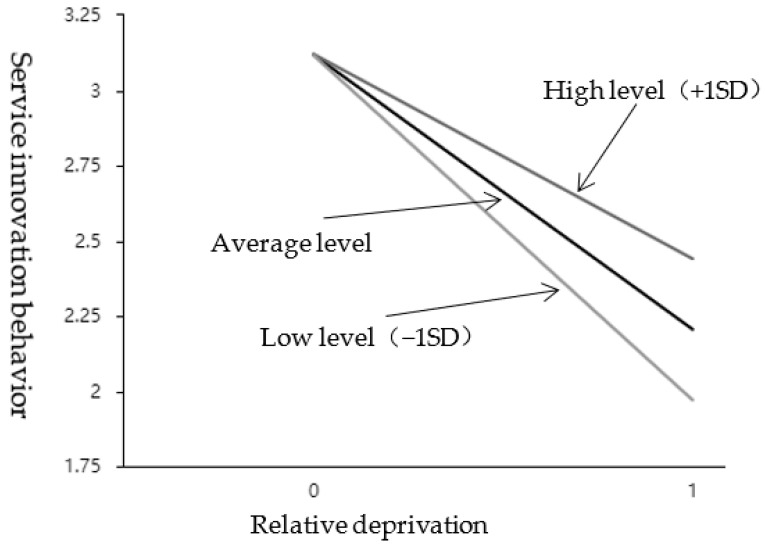
The effect of interaction between RD and IAS on SIB.

As shown in Table 7, the interaction between relative deprivation and internal attribution style showed a significant (t = 4.255, *p* = 0.000 < 0.05), and it was a positive effect (β = 0.229, *p* < 0.05). It means that when relative deprivation affects service innovation behavior, the moderation variable (internal attribution style) has a significant difference in the extent of influence at different levels. See the simple slope diagram in Figure 4. Divide the scores of the internal attribution style into two groups according to high (M + 1SD) and low (M − 1SD). Perform a simple slope analysis. As shown in Figure 4, the top line represents the relationship between relative deprivation and service innovation under a high internal attribution style; the bottom line represents the relationship between relative deprivation and service innovation behavior under a low internal attribution style; the middle line is the average level. It can be seen that the attributive style of employees’ internal is at a low level, and the relationship curve between relative deprivation and employees’ service innovation behavior is steeper and the slope is bigger; when the employee internal attribution style is at a high level, the relationship curve between relative deprivation and employees’ service innovation behavior is gentler, and the slope is smaller. The negative regulatory effect of internal control attribution was further tested; that is, H3b was further supported by empirical data.

#### 4.3.4. Moderated Mediating Effect Testing

As shown in Table 8, the Bootstrapping method is used to analyze the intermediary effect of the relative deprivation between organizational justice and service innovation behavior under different levels of external attribution style. The specific method is to add or subtract a standard deviation from the mean value of the external attribution style, divide the scores into two groups according to high (M + 1SD) and low (M − 1SD), obtain the values of the external attribution style at two different levels, and calculate the intermediary effect of relative deprivation at these two different levels. For the intermediary variable of relative deprivation, when the level is low, the boot 95% CI includes the number 0, which means there is no mediation at this level. At the average level, the boot 95% CI does not include the number 0, which means that it has a mediating effect at this level, and the Effect value is 0.420. At a high level, the boot 95% CI does not include the number 0, which means it has an intermediary effect at this level, and the Effect value is 0.556. To summarize, we can see that at different levels, the intermediary role is inconsistent, indicating that it has a moderated mediating role. Therefore, H4a is verified.

As shown in Table 9, the Bootstrapping method is used to analyze the intermediary effect of the relative deprivation between organizational justice and service innovation behavior under different levels of internal attribution style. The specific method is to add or subtract a standard deviation from the mean value of the internal attribution style, divide the scores into two groups according to high (M + 1SD) and low (M − 1SD), obtain the values of the internal attribution style at two different levels, and calculate the intermediary effect of relative deprivation at these two different levels. For the intermediary variable of relative deprivation, when the level is low, the boot 95% CI does not include the number 0, which means that there is an intermediary effect at this level, and the Effect value is 0.726. At the average level, the boot 95% CI does not include the number 0, which means that it has an intermediary effect at this level, and the Effect value is 0.545. At a high level, the boot 95% CI includes the number 0, which means there is no mediation at this level. To sum up, we can see that at different levels, the intermediary role is inconsistent, indicating that it has a moderated mediating role. Therefore, H4b is verified.

## 5. Conclusions and Discussion

### 5.1. Conclusions

For service-oriented enterprises, their employees are often faced with changing working environments and uncertain working conditions, which have higher requirements for flexibility and the innovation abilities of employees. In the new era, being able to take service innovation behavior in the work process has become a necessary quality for employees of service-oriented enterprises. The management experience of service-oriented enterprises also emphasizes the importance of the internal environment of the organization. From the perspective of employees in service-oriented enterprises, this paper discusses the impact of organizational internal environment perception on employee work behavior, and the impact of organizational fairness on service innovation behavior is highly practical. Therefore, this study starts from the perspective of employees in service-oriented enterprises. To explore whether the service innovation behavior of employees in service-oriented enterprises is affected by organizational fairness and further identify the role of relative deprivation and attribution style. The findings are as follows: (1) Organizational fairness has a significant positive predictive effect on employee service innovation behavior. (2) Relative deprivation plays a part of the intermediary role between organizational fairness and employee service innovation behavior. (3) The external attribution style has a significant positive moderation effect on the relationship between relative deprivation and employee service innovation behavior. The more employees tend to external attributional style, the stronger the negative relationship between the relative deprivation and employee service innovation behavior. The internal attribution style has a significant negative moderation effect on the relationship between relative deprivation and employees’ service innovation behavior. The more employees tend to internal attributional style, the weaker the negative relationship between the relative deprivation and employees’ service innovation behavior. (4) Employees’ external attribution style has a significant positive moderation effect on the indirect relationship between organizational fairness and employees’ service innovation behavior through relative deprivation. The more employees tend to external attribution style, the stronger the negative indirect relationship. Employees’ internal attribution style has a significant negative moderation effect on the indirect relationship between organizational fairness and employees’ service innovation behavior through relative deprivation. The more employees tend to internal attribution style, the weaker the negative indirect relationship.

### 5.2. Discussion

#### 5.2.1. Theoretical Implications

This study confirmed the predictive effect of organizational fairness on the service innovation behavior of employees in service-oriented enterprises. The existing scholars pay attention to the consequences of organizational fairness, ignoring the impact of organizational fairness on the service innovation behavior of employees in service-oriented enterprises. In view of this, this research is based on a questionnaire survey, taking employees of service-oriented enterprises as the research object, and starting from the factors of organizational fairness, it further deepens the cognition of the relationship between organizational fairness and service innovation behavior of employees in service-oriented enterprises. This study supplements the research results between organizational fairness and service innovation behavior as one of the perceptions of the internal environment of the organization. To some extent, this conclusion supports Xu’s conclusion that organizational fairness has a significant positive impact on employee satisfaction [16] and G Wang’s conclusion that the organizational fairness has a positive impact on organizational citizenship behavior of employees in private enterprises [24]. It provides a theoretical explanation for the service innovation behavior of employees in service-oriented enterprises.

Second, the introduction of relative deprivation as a mediator has explored the mechanism between organizational fairness and the service innovation behavior of employees in service-oriented enterprises, supplemented the content of the research on the intermediary mechanism of service innovation behavior through the path of individual cognition and emotional experience of the internal environment of the organization, which is conducive to a deeper understanding of the impact of organizational fairness on employees in service-oriented enterprises. Different from previous studies, this study conducted an analysis from the perspective of social cognitive theory, providing an effective analytical framework for exploring the mechanism of organizational fairness on the service innovation behavior of employees in service-oriented enterprises. On the individual level, a low level of organizational fairness will damage the enthusiasm of employees, cause work problems and pressure, adversely affect work enthusiasm, and may also affect interpersonal relationships within the organization. On the organizational level, the unfair internal environment of the organization will destroy the positive organizational atmosphere, greatly reduce the enthusiasm of employees at two levels, and ultimately lead to reduced employee service innovation behavior.

Third, this study tested the moderation effect of the attribution style of employees in service-oriented enterprises. Different attribution styles will have an impact on the subsequent behavioral responses of employees who have a sense of relative deprivation. Compared with employees who prefer internal attribution style, employees who are relatively inclined to the external attribution style will have a stronger emotional and behavioral response after having a sense of relative deprivation and are more likely to make negative feedback on the cognition of others and organizational situations, these negative perceptions and reactions will aggravate the negative impact of relative deprivation on service innovation behavior [58]. Employees’ sense of organizational fairness in the workplace will decline more significantly, which will further inhibit employee service innovation behavior. On the one hand, this study provides empirical support for the interaction model of employees’ relative deprivation; on the other hand, it also deeply explores the boundary of attribution in service-oriented enterprises; that is, the interaction between attribution style, individual psychology, and organizational fairness in employee service innovation behavior. This study is an important supplement and extension to the research on organizational internal environment perception and service innovation behavior.

#### 5.2.2. Management Implications

The results show that organizational fairness has a significant positive impact on employee service innovation behavior, which provides a new way for service-oriented enterprises to stimulate their employee service innovation behavior. As an important perception of the internal environment of an organization, organizational justice has an important impact on the enthusiasm and behavior of employees and is of great significance to the business performance and long-term development of an enterprise. Therefore, service-oriented enterprises should pay attention to maintaining the internal environment of organizational fairness, improve the level of employees’ sense of organizational fairness, take measures from the source, and establish a fair management system. At the same time, service-oriented enterprises should ensure the fairness of procedures, improve the supervision mechanism, ensure the universality of decisions and systems, be open and transparent in the implementation process, provide employees with channels to reflect on problems, put forward appeals, and accept the supervision of employees. In addition, management communication should be strengthened, and communication channels between superiors and subordinates should be broadened. Enterprises should establish a sound internal communication mechanism, such as holding round tables and establishing complaint mailboxes, to achieve effective internal communication. Managers should also pay attention to their own communication methods and attitudes in the process of communicating with employees, provide employees with their due rights and respect, and create a good internal organizational environment, improving employees’ sense of organizational fairness and organizational cohesion.

Second, the research shows that the sense of relative deprivation plays a mediation role between the sense of organizational fairness and employee service innovation behavior, which reveals that employees’ low-level sense of organizational fairness will lead to an increase in the employees’ sense of relative deprivation, which will have a negative impact on employee service innovation behavior. In daily management, managers need not only to play the role of leaders but also to play the role of communicators. They should pay attention to the implementation of humanistic management, strengthen the interaction and emotional communication with employees, pay attention to the emotional changes of employees in a timely manner, and conduct emotional counseling for employees with negative emotions in a timely manner, so as to effectively avoid the occurrence of the sense of relative deprivation of employees, thus promoting the service innovation behavior of employees. Research shows that by enhancing the sense of belonging [59], fairness of procedures [60], advance notice of news [61], and group identity [62], the relative deprivation of employees can be reduced significantly. Therefore, for service-oriented enterprises, to prevent the occurrence of the sense of relative deprivation of employees, they can reduce the sense of relative deprivation of employees by implementing a reasonable labor relations system, ensuring the fairness and reasonableness of distribution and promotion mechanisms, ensuring the openness and transparency of information in the organization, humanized management of managers, training of employees, and other measures to stimulate employee service innovation behavior.

Third, the study found that external attribution style can enhance the direct negative relationship between employee relative deprivation and service innovation behavior and the intermediary effect of relative deprivation in the relationship between organizational fairness and employee service innovation behavior, while an internal attribution style can weaken the direct negative relationship between employee relative deprivation and service innovation behavior and the intermediary effect of relative deprivation in the relationship between organizational fairness and employee service innovation behavior. This inspires enterprise managers to pay attention to the screening of employees’ personality characteristics in the recruitment process, give priority to individuals with strong emotional management ability and inclination to internal attribution style, and maintain employees’ high work passion through various ways to reduce the impact of unfair perception and relative deprivation of the organization’s internal environment on their service innovation behavior. Meanwhile, in daily training, consciously guide and educate employees. When negative emotions and bad perceptions occur, guide employees to analyze and solve problems with a more inclusive attitude and an objective and rational attitude so as to jointly achieve win–win development of enterprises and individuals. In the context of the normalization of the global COVID-19 pandemic, service-oriented enterprises are facing many challenges. The managers of service-oriented enterprises need to cultivate a group of employees with strong emotional management ability and focus on strengthening their perception of positive events so as to expand the positive significance of positive events and stimulate their positive work behavior [63]. At the same time, it should be noted that attribution is a double-edged sword, and employees’ personality characteristics have both advantages and disadvantages in work. The boundary of attribution in different problems is different [64,65,66]. In the process of solving practical problems, managers need to deal with specific problems.

#### 5.2.3. Limitations and Suggestions for Future Research

First of all, this study takes employees of service-oriented enterprises as the research subject, and the samples are mainly concentrated in Shanxi. Whether the research conclusions are applicable to other regions and other industries remain to be further verified. Future research can focus on different samples across regions and industries. Secondly, the impact of organizational fairness on employee service innovation behavior is a complex process. This paper only considers the establishment of a model from two aspects of relative deprivation and attribution style. Future research can introduce more variables to explore the impact mechanism of organizational fairness on service innovation behavior from different theoretical perspectives. Finally, this study only explores the service innovation behavior of individuals but also needs to explain the impact of organizational fairness on individual behavior and the entire organization. Therefore, future research can build a multi-level model between organizational fairness and organizational innovation to explore the impact of organizational fairness on employee behavior and on internal knowledge management and knowledge innovation.

## Figures and Tables

**Table 1:** Reliability and aggregation validity of the scale.

Constructs	Items	Std. Estimate	z (CR)	Cronbach’s α	AVE
Organizational fairness	Z1	0.923	0.923	0.92	0.857
	Z2	0.921			
	Z3	0.938			
	Z4	0.943			
	Z5	0.912			
	Z6	0.917			
Relative deprivation	X1	0.881	0.876	0.866	0.759
	X2	0.852			
	X3	0.887			
	X4	0.864			
Service innovation behavior	F1	0.898	0.895	0.883	0.682
	F2	0.88			
	F3	0.693			
	F4	0.818			
Internal attribution style	W1	0.812	0.773	0.77	0.552
	W2	0.683			
	W3	0.758			
	W4	0.712			
External attribution style	N1	0.831	0.719	0.723	0.534
	N2	0.723			
	N3	0.667			
	N4	0.691			

**Table 2:** Correlation coefficients and discriminant validity.

	Average Value	SD	Gender	Age	Education	Working Years	Organizational Fairness	Relative Deprivation	Service Innovation Behavior	Internal Attribution Style	External Attribution Style
Gender	1.482	0.501									
Age	2.68	1.217	0.105								
Education	3.194	0.694	0.107	0.327							
Working years	2.755	0.879	0.053	0.271 **	0.123 **						
Organizational fairness	3.536	0.989	−0.007	−0.029	0.042	−0.081	0.925				
Relative deprivation	2.564	0.989	0.021	0.247	−0.027	0.169	−0.923 **	0.871			
Service innovation behavior	3.481	1.045	0.017	−0.092	−0.055	−0.176 **	0.846 **	−0.846 **	0.826		
Internal attribution style	2.404	0.821	0.031	0.123	−0.009	0.05	0.035	0.076	0.019	0.743	
External attribution style	3.76	0.708	0.065	−0.185 **	−0.056	−0.144 *	0.406 **	−0.329 **	0.407 **	−0.320 **	0.731

Note: Diagonal values are square roots of AVE and values below the diagonal are Pearson correlation coefficients between dimensions; * *p* < 0.05; ** *p* < 0.01.

**Table 3:** Path coefficients.

X	→	Y	Non-Standardized Path	SE	z (CR)	*p*	Standardized Path
Organizational fairness	→	Relative deprivation	−0.88	0.038	−23.421	0	−0.923
Organizational fairness	→	Service innovation behavior	0.446	0.137	3.258	0.001	0.439
Relative deprivation	→	Service innovation behavior	−0.47	0.144	−3.269	0.001	−0.441

**Table 4:** Mediating effects model tests.

	Service Innovation Behavior	Relative Deprivation	Service Innovation Behavior
Constant	0.442 * (2.366)	5.686 ** (44.940)	3.111 ** (3.664)
Organizational fairness	0.859 ** (15.324)	−0.880 ** (−23.174)	0.446 ** (3.207)
Relative deprivation			−0.469 ** (−3.215)
R 2	0.716	0.852	0.745
Adjust R2	0.713	0.851	0.739
F	F (1,93) = 234.815, *p* = 0.000	F (1,93) = 537.018, *p* = 0.000	F (2,92) = 134.365, *p* = 0.000

Note: T-value in parentheses; * *p* < 0.05; ** *p* < 0.01.

**Table 5:** Mediation effects test results.

Path	c Total Effect	a	b	a × b	a × b (Boot SE)	a × b (z)	a × b (*p*)	a × b (95% BootCI)	c’	Test Conclusion
Organizational fairness →Relative deprivation →Service innovation behavior	0.859 **	−0.880 **	−0.469 **	0.413	0.151	2.742	0.006	0.018 ~ 0.666	0.446 **	Partial mediation

Note: ** *p* < 0.01.

**Table 6:** Moderating effect test results1.

	Model 1					Model 2					Model 3				
	B	SE	t	*p*	β	B	SE	t	*p*	β	B	SE	t	*p*	β
Constant	3.137	0.063	49.705	0.000 **	-	3.137	0.061	51.16	0.000 **	-	3.087	0.064	48.386	0.000 **	-
Relative deprivation	−0.902	0.059	−15.329	0.000 **	−0.846	−0.851	0.061	−14.063	0.000 **	−0.799	−0.848	0.059	−14.33	0.000 **	−0.796
External attribution style						0.28	0.11	2.555	0.012 *	0.145	0.399	0.119	3.352	0.001 **	0.207
Relative deprivation * External attribution style											−0.241	0.105	−2.298	0.024 *	−0.135
R 2	0.716					0.735					0.75				
Adjust R 2	0.713					0.729					0.742				
F	F (1,93) = 234.975, *p* = 0.000					F (2,92) = 127.732, *p* = 0.000					F (3,91) = 90.880, *p* = 0.000				
ΔR 2	0.716					0.019					0.015				
ΔF	F (1,93) = 234.975, *p* = 0.000					F (1,92) = 6.526, *p* = 0.012					F (1,91) = 5.283, *p* = 0.024				

Note: Dependent variable: service innovation behavior; * *p* < 0.05; ** *p* < 0.01.

**Table 7:** Moderating effect test results2.

	Model 1					Model 2					Model 3				
	B	SE	t	*p*	β	B	SE	t	*p*	β	B	SE	t	*p*	β
Constant	3.137	0.063	49.705	0.000 **	-	3.137	0.063	50.06	0.000 **	-	3.118	0.058	54.03	0.000 **	-
Relative deprivation	−0.902	0.059	−15.329	0.000 **	−0.846	−0.908	0.059	−15.51	0.000 **	−0.853	−0.913	0.054	−16.966	0.000 **	−0.857
Internal attribution style						0.126	0.083	1.527	0.13	0.084	0.003	0.081	0.041	0.968	0.002
Relative deprivation * Internal attribution style											0.304	0.071	4.255	0.000 **	0.229
R 2	0.716					0.723					0.769				
Adjust R 2	0.713					0.717					0.762				
F	F (1,93) = 234.975, *p* = 0.000					F (2,92) = 120.338, *p* = 0.000					F (3,91) = 101.175, *p* = 0.000				
ΔR 2	0.716					0.007					0.046				
ΔF	F (1,93) = 234.975, *p* = 0.000					F (1,92) = 2.333, *p* = 0.130					F (1,91) = 18.104, *p* = 0.000				

Note: Dependent variable: service innovation behavior; * *p* < 0.05; ** *p* < 0.01.

**Table 8:** Conditional indirect effect test results 1.

Intermediary Variable	Level	Level Value	Effect	BootSE	BootLLCI	BootULCI
Relative deprivation	Low level (−1SD)	3.095	0.283	0.199	−0.098	0.681
	Average level	3.689	0.42	0.153	0.12	0.732
	High level (1SD)	4.284	0.556	0.141	0.256	0.844

**Table 9:** Conditional indirect effect test results 2.

Intermediary Variable	Level	Level Value	Effect	BootSE	BootLLCI	BootULCI
Relative deprivation	Low level (−1SD)	1.582	0.726	0.192	0.308	1.063
	Average level	2.347	0.545	0.172	0.119	0.815
	High level (1SD)	3.113	0.363	0.191	−0.119	0.64

## Data Availability

The data analyzed in this paper are proprietary and, therefore, cannot be posted online.
